# Supplementary material for: Effects of Dietary Linoleic Acid on Blood Lipid Profiles: A Systematic Review and Meta-Analysis of 40 Randomized Controlled Trials
Source: Foods. 2023 May 25;12(11):2129. doi: 10.3390/foods12112129 (PMC10253160; doi:10.3390/foods12112129)
Supplement: Supplementary file 1 [file foods-12-02129-s001.zip › foods-2360780-supplementary.pdf]

Table S1 Quality assessment of the included studies using Cochrane.

| Study | Random sequence generation | Allocation concealment | Blinding of participants and personnel | Blinding of outcome assessments | Incomplete outcome data | Selective outcome reporting | Other bias |
|---|---|---|---|---|---|---|---|
| Abbey et al. (1990) | U | U | L | U | L | U | L |
| Aguilera et al. (2004) | L | U | U | U | L | L | L |
| Akrami et al. (2018) | L | U | U | U | L | L | L |
| Atefi et al. (2018) | L | U | U | U | L | L | L |
| Bjermo et al. (2012) | U | U | U | U | L | U | L |
| Brassard et al. (2018) | U | U | U | U | U | L | L |
| Cândido et al. (2018) | L | L | L | L | L | U | L |
| Cicero et al. (2009) | U | U | L | U | U | U | L |
| Damsgaard et al. (2008) | L | L | L | L | L | L | L |
| Dittrich et al. (2015) | U | U | L | U | L | U | L |
| Goyens et al. (2005) | U | U | L | U | L | U | L |
| Han et al. (2012) | U | U | L | L | L | U | L |
| Iggman et al. (2014) | U | U | L | U | L | L | L |
| Jones et al. (2014) | L | U | L | U | L | L | L |
| Jones et al. (2015) | U | U | L | U | L | L | L |
| Junker et al. (2001) | U | U | U | U | L | L | L |
| Karakas et al. (2016) | U | U | L | U | L | L | L |
| Karupaiah et al. (2016) | U | U | L | U | L | U | L |
| Kaul et al. (2008) | L | U | L | U | U | U | L |
| Kawakami et al. (2015) | U | U | L | U | L | U | L |
| Lee et al. (2012) | L | U | U | U | L | U | L |
| Lichtenstein et al. (1993) | L | U | L | U | L | L | L |
| Lv et al. (1993) | U | U | H | U | L | L | L |

| Study | Random sequence generation | Allocation concealment | Blinding of participants and personnel | Blinding of outcome assessments | Incomplete outcome data | Selective outcome reporting | Other bias |
|---|---|---|---|---|---|---|---|
| Nigam et al. (2014) | L | U | U | U | L | U | L |
| Oliveira-de-Lira et al. (2018) | U | L | L | U | L | L | L |
| Pang et al. (1998) | U | U | U | U | U | U | L |
| Paschos et al. (2007) | U | U | H | U | U | U | L |
| Pu et al. (2016) | U | U | L | U | L | L | L |
| Rallidis et al. (2003) | U | U | U | U | U | U | L |
| Rezaei et al. (2019) | L | L | L | U | L | L | L |
| Rozati et al. (2015) | U | L | H | U | L | U | L |
| Salar et al. (2006) | L | U | H | U | L | L | L |
| Scholtz et al. (2004) | U | U | H | H | L | U | L |
| Stricker et al. (2008) | L | U | L | U | L | U | L |
| Ulven et al. (2016) | U | U | L | U | L | L | L |
| Vafeiadou et al. (2015) | L | U | H | H | L | L | L |
| Vargas et al. (2011) | U | U | L | U | L | U | L |
| Wilkinson et al. (2005) | U | U | H | U | U | U | L |
| Yang et al. (2019) | L | U | L | U | L | L | L |
| Zheng et al. (2016) | L | U | L | U | L | L | L |

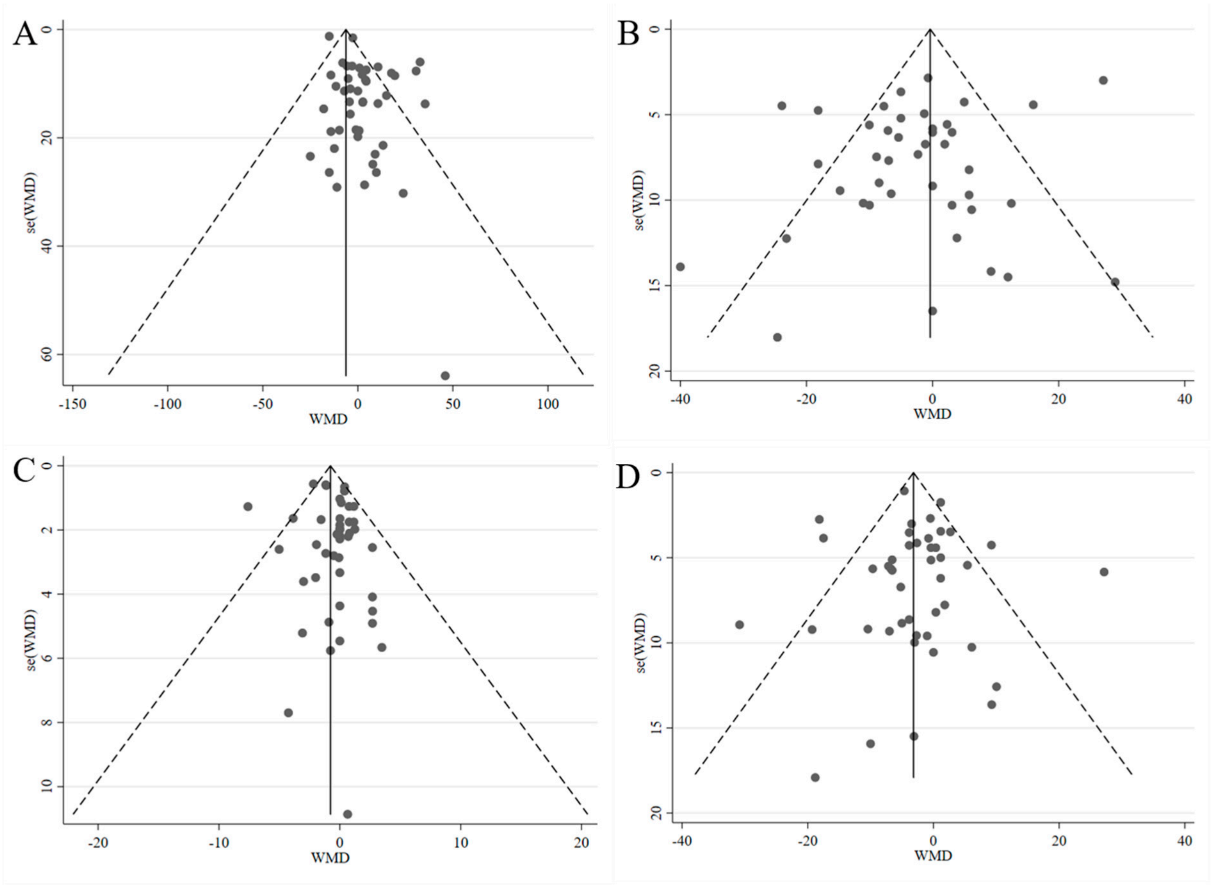


Figure S1 Funnel plots of LA consumption and TG (A), TC (B), HDL-C (C) and LDL-C (D).

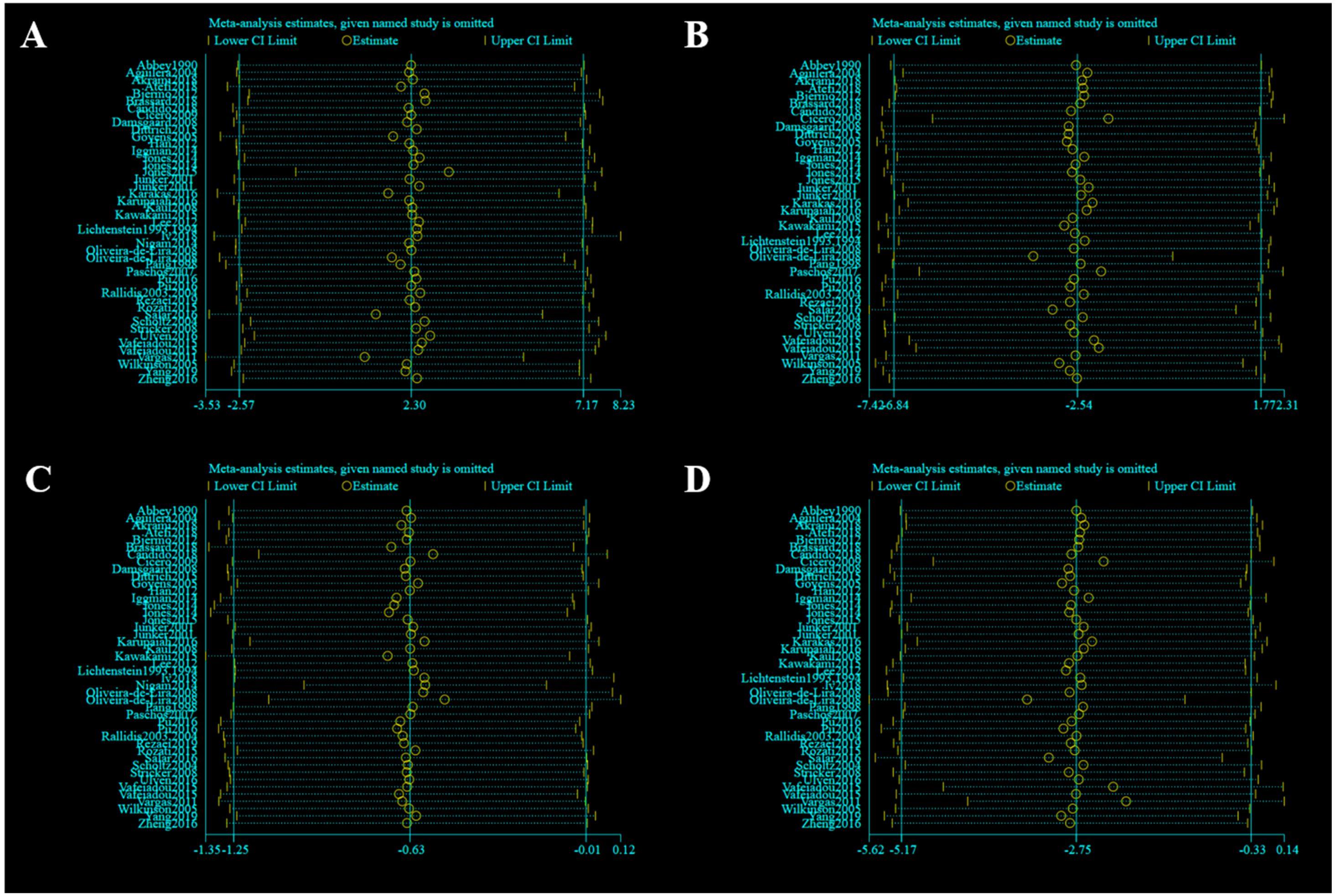


Figure S2 Sensitivity analysis of LA consumption and TG (A), TC (B), HDL-C (C) and LDL-C (D).
